# Efficacy and safety of everolimus in heart transplant recipients: A meta-analysis

**DOI:** 10.1016/j.jhlto.2026.100610

**Published:** 2026-07-21

**Authors:** Sofia Helena Vitte, Antonio Medina Luna, Julia Jersak Pille, Bruno Nazário Pinheiro, Marcella Baggio Lopes de Souza, Clariphen Debbie Joseph Irudhayaraj, Giancarlo Maruri Munaretto

**Affiliations:** a Hospital de Amor Nossa Senhora, Barretos, Brazil; b Universidad de Monterrey Division de Ciencias de la Salud, México, México; c University of the Extreme South of Santa Catarina, Santa Catarina, Brazil; d Faculdade Santa Marcelina, São Paulo, Brazil; e PSG Institute of Medical Sciences and Research, Coimbatore, Tamil Nadu, India; f Universidade do Contestado - Campus Concordia, Santa Catarina, Brazil

**Keywords:** Cardiac surgery, Heart transplant, Immunosuppression, Toxicity, Cardiac allograft vasculopathy

## Abstract

**Background:**

Long-term outcomes after heart transplantation remain limited by cardiac allograft vasculopathy (CAV) and calcineurin inhibitor (CNI)–related nephrotoxicity. Everolimus has emerged as a CNI-sparing strategy, yet its net clinical benefit remains debated.

**Objectives:**

To determine whether everolimus-based immunosuppression improves efficacy and safety compared with standard CNI-based therapy in heart transplant recipients.

**Methods:**

We conducted a systematic review and meta-analysis of randomized controlled trials and comparative observational studies enrolling adult and/or pediatric heart transplant recipients. MEDLINE, EMBASE, and CENTRAL were searched from inception. The primary endpoint was the composite MATE-3 (acute rejection, CAV, or chronic kidney disease). Secondary outcomes included mortality, individual efficacy components, renal function, and the broader safety composite (MATE-6). Pooled effect estimates were generated using random-effects models.

**Results:**

Sixty studies including 1786 heart transplant recipients (mean age ≈52 years) were analyzed. Everolimus-based regimens reduced cardiac allograft vasculopathy progression by intravascular ultrasound (RR ≈0.57) and improved renal function when combined with CNI minimization (mean eGFR increase ≈10–15 mL/min). The composite MATE-3 endpoint favored everolimus. All-cause mortality was neutral (RR ≈0.98). Acute rejection varied by protocol, with higher rates mainly in strategies involving abrupt or late CNI withdrawal. The safety composite (MATE-6) showed increased adverse events and treatment discontinuation with everolimus.

Sixty studies were included. Everolimus-based regimens significantly reduced CAV progression by intravascular ultrasound and consistently preserved renal function when coupled with CNI minimization. The composite MATE-3 endpoint favored everolimus, driven by robust vascular and renal protection. Importantly, all-cause mortality was neutral, confirming that CNI reduction with everolimus does not compromise survival. Acute rejection demonstrated protocol-dependent variability, with excess risk observed primarily in strategies involving abrupt or late CNI withdrawal. The safety composite (MATE-6) revealed higher rates of drug discontinuation and adverse events, underscoring the need for careful monitoring.

**Conclusions:**

Everolimus-based immunosuppression provides meaningful vasculoprotection and renal preservation without increasing mortality. When used within structured CNI minimization protocols, it offers a strategy to improve long-term graft health after heart transplantation. Careful and individualized use is essential to balance efficacy and tolerability.

## Introduction

Since the first heart transplantation in 1967, advances in surgical techniques and immunosuppressive strategies have substantially improved survival, with more than 104,000 heart transplants performed worldwide and median post-transplant survival approaching 11 years in the contemporary era.^1^ The introduction of calcineurin inhibitors (CNIs) in the early 1980s represented a major milestone, significantly reducing acute rejection and early graft loss. Nevertheless, long-term outcomes remain limited by chronic complications, particularly cardiac allograft vasculopathy (CAV), which affects up to 50% of heart transplant recipients within five years and remains a leading cause of late mortality.^1^, ^2^, ^3^

Despite their efficacy, CNIs are associated with substantial toxicities, including nephrotoxicity, metabolic derangements, and promotion of CAV progression. These adverse effects affects both adult and pediatric recipients and are particularly consequential in younger patients due to lifelong exposure to immunosuppression. Achieving an optimal balance between preventing rejection and minimizing long-term toxicity therefore remains a central challenge in heart transplantation across age groups.^1^, ^4^

Mammalian target of rapamycin (mTOR) inhibitors, such as everolimus, have emerged as CNI-sparing agents that allow dose reduction while maintaining immunologic control. Studies in heart transplantation suggest that everolimus-based regimens may attenuate CAV progression and preserve renal function without increasing the risk of acute rejection. However, the available evidence remains heterogeneous, largely derived from small trials and observational cohorts, and has not been systematically synthesized in populations including both adult and pediatric recipients.^2^

We hypothesized that, among heart transplant recipients who are clinically stable at six months post-transplant, an immunosuppressive strategy combining everolimus with low-dose tacrolimus would be non-inferior or superior to standard-dose tacrolimus plus mycophenolate mofetil or other conventional regimens in terms of efficacy, while offering a favorable safety profile. Accordingly, this systematic review and meta-analysis aimed to quantitatively compare these strategies with respect to a composite efficacy outcome, including acute rejection, cardiac allograft vasculopathy, renal dysfunction, and safety outcomes.

**Table 1:** Baseline of Population

Study	Design	Patients EVR/CTRL	Males, %	Time since transplant^a^	Age^†^, y	BMI^c^	Renal function^b^	IVUS/CAV	MATE-3, %	MATE-6, %	Main underlying cause for transplant	Type of graft: first or retransplantation
SCHEDULE TRIAL	RCT	49/51	76.8%/78%	Late	50+14/51+11	26.5±4.2; 26.6±3.3.	82.7 mL/min / 61.0 mL/min	0.09+-0.05/0.15+-0.16	7.1% / 5.1%	37.3%/ 19.6%	Dilated cardiomyopathy	First
TEAMMATE TRIAL	RCT	107/104	51%/58%	Early	13.4+5.2/12.9+5,6	0.6+1.1/0.5+1.1	102.5+-20.4/ 97.8+-22.1	NR	18.7% /16.3%	89.7%/58.7%	Dilated cardiomyopathy	First
MANDELA STUDY	RCT	78/84	83.1%/82.4%	Early	51.4+11.4/51.4+10.2	25.7±3.8 25.3±3.2	EVR: +10.2 mL/min (Gain)→CTRL: +1.4 mL/min (Stable)"	0,02 mm /0.03 mm	12.1%/ 2.8%	9.1%/5.6%	Dilated cardiomyopathy	First
SHIRAKISS TRIAL	RCT	88/101	80/87%	Late	55.4+9.7/50.3+11.9	25.2+4.1/25.1+3.1	61.7+-20.4/ 48.9+-14	NR	0.03 +/-0.05 mm | 0.07 +-0.11 mm	18.2%/27,3%	Dilated cardiomyopathy	
CERTICAN TRIAL^d^	RCT	17/17	72.5%/74.3%	Early	51.1+10.8/51.4+11.2	25.4±4.2/ 25.6±4.5	141.4 μmol/L /114.0 μmol/L (Cr)	0.08 mm / /0.12 mm	31.7%/ 46.7%	25.0%/ 6.5%	Ischemic cardiomyopathy	First
RAD-TAC STUDY	RCT	28/25	82.6%/80.9%	Late	52+11/51+12	25.8+3.6/26+4.7	65.5+-18.3/56.7+-15.2	NR	16.7%/20.8%	16.7%/25%	Dilated cardiomyopathy	First
TRANSFORM STUDY	RCT	17/17	82%/77%	Late	51.1+12.5/51+12.7	26.2+4.7/26.5+4.8	57+/- 15 /46+/- 14	NR	24.2%/24.6%	29.4%/40.2%	Dilated cardiomyopathy	First
KOTRY STUDY	COHORT	144/478	64.8%/73.3%	Late	54.8+11.2/56.2+12.5	23.4 ± 3.4 kg/m²/24.5 ± 3.6 kg/m²	72.3 | 75.8	NR	12.7% vs 18.6%	23.9%/24.4%	Dilated cardiomyopathy	First
Massetti, 2013^5^	RCT	50/50	94.7%/85%	Late	55.4+9.7/50.3+11.9	25.3±3.4 kg/m² | 24.1 ± 3.2 kg/m²	54.4 | 53.6	0.03±0.05 mm/ 0.10 ± 0.11 mm	5.3% | 0.0%	26.3% | 10.0%	Ischemic cardiomyopathy	First
CERCARI TRIAL	RCT	50/50	79.3%/89.3%	Late	61+14.9/58.3+11.5	26.2±4.1 kg/m² | 27.1 ± 3.8 kg/m².	+9.4 mL/min /+1.9 mL/min	0.02+-0.08/0.01+-0.08	6.9% | 7.1%	41.4% | 7.1%	Dilated cardiomyopathy	First
Chou, 2007^6^	RCT	16/8	87.5%/75%	Late	55.6+9.1/46.4+9.3	NR	NR	NR	8.3%/16.7%	16.7%/25%	Dilated cardiomyopathy	First
Watanabe, 2013^7^	RCT	24/39	90%/82%	Late	51+12/48+13	23.4+2.9/23.7+2.8	54.6+-14.3/51.3+-13.8	NR	9.5%/14.3%	14.3%/19%	Dilated cardiomyopathy	First
Stypmann, 2015^8^	RCT	48/34	87.5%/73,5%	Late	55.4+9.1/51.6+11.8	NR	48.95 / 58.60	NR	12.5%/18.7%	14.6%/22.9%	Isquemic cardiomyopathy	First
NOCTET TRIAL	RCT	19/20	68.4%/70%	Late	56.6+10.5/60.9+7.8	25.7±4.6/26.8 ± 4.1	NR	NR	4,3%/2.8%	12.1%/16.9%	Dilated cardiomyopathy	First
Schweiger, 2006^9^	RCT	15/14	86.7%/85.7%	Late	51+14/48+14	NR	68+18/62+15	NR	10%/15%	22.5%/30%	Ischemic cardiomyopathy	First

Table 1. Baseline characteristics of included full-paper studies.

^†^mean or median; EVR: Everolimus; CTRL: Control; NR: not reported; RCT: randomized controlled trial;.

a early (<12months), late (>12 months);

b mean+-sd or gain in eGFR;

c sd+-mean or BMI-z escore;

d due to 2 intervention groups, results presented are the pooled combined data

## Methods

### Study design

**Figure 1:**
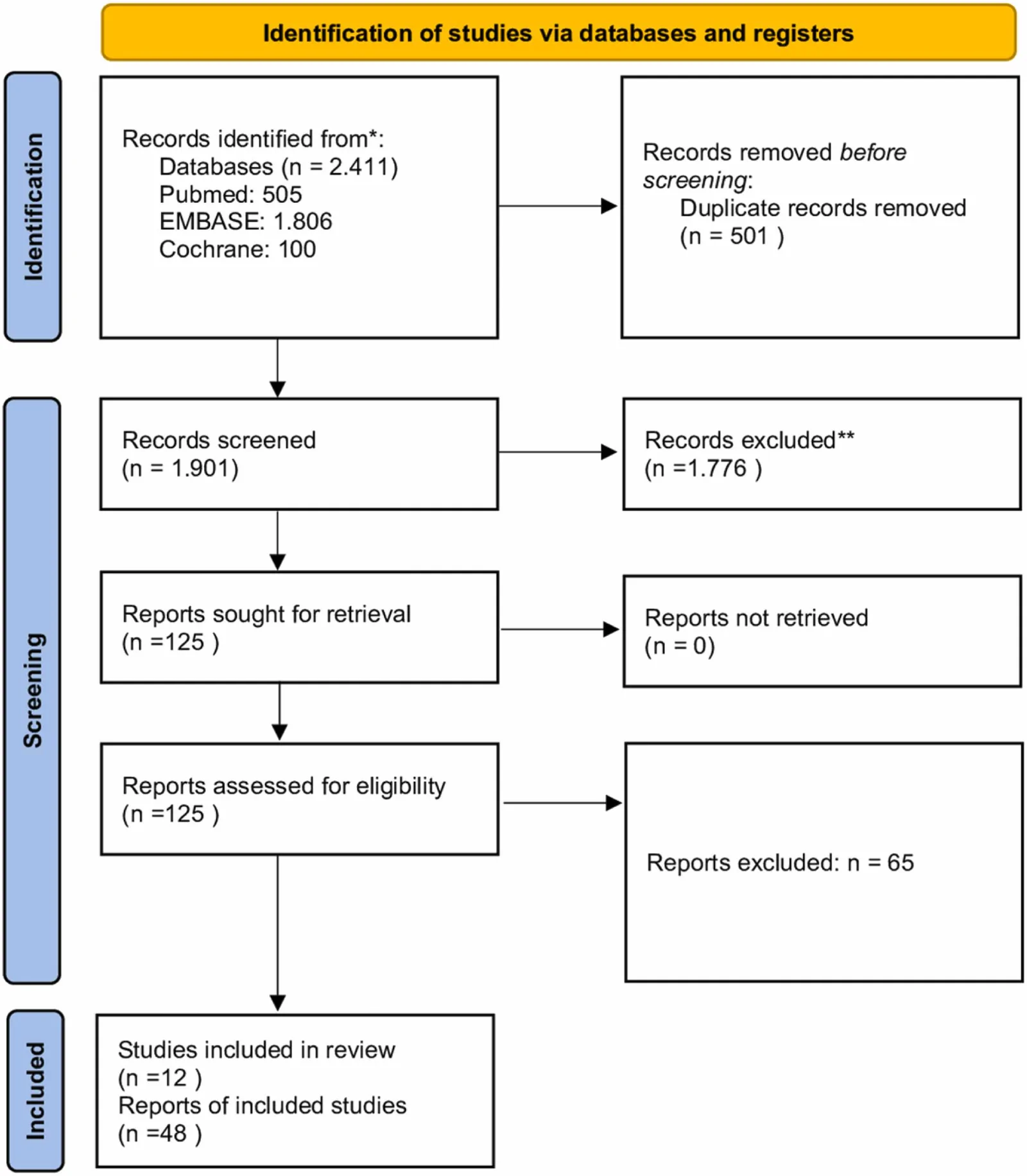
PRISMA flow chart.

This systematic review and meta-analysis was conducted in accordance with the Cochrane Handbook for Systematic Reviews of Interventions and reported following the Preferred Reporting Items for Systematic Reviews and Meta-Analyses (PRISMA) guidelines (Figure 1). The study protocol was prospectively registered in the International Prospective Register of Systematic Reviews (PROSPERO; registration number **CRD420261292356**). The complete PRISMA checklist is provided in the Supplementary Materials.

**Figure 2:**
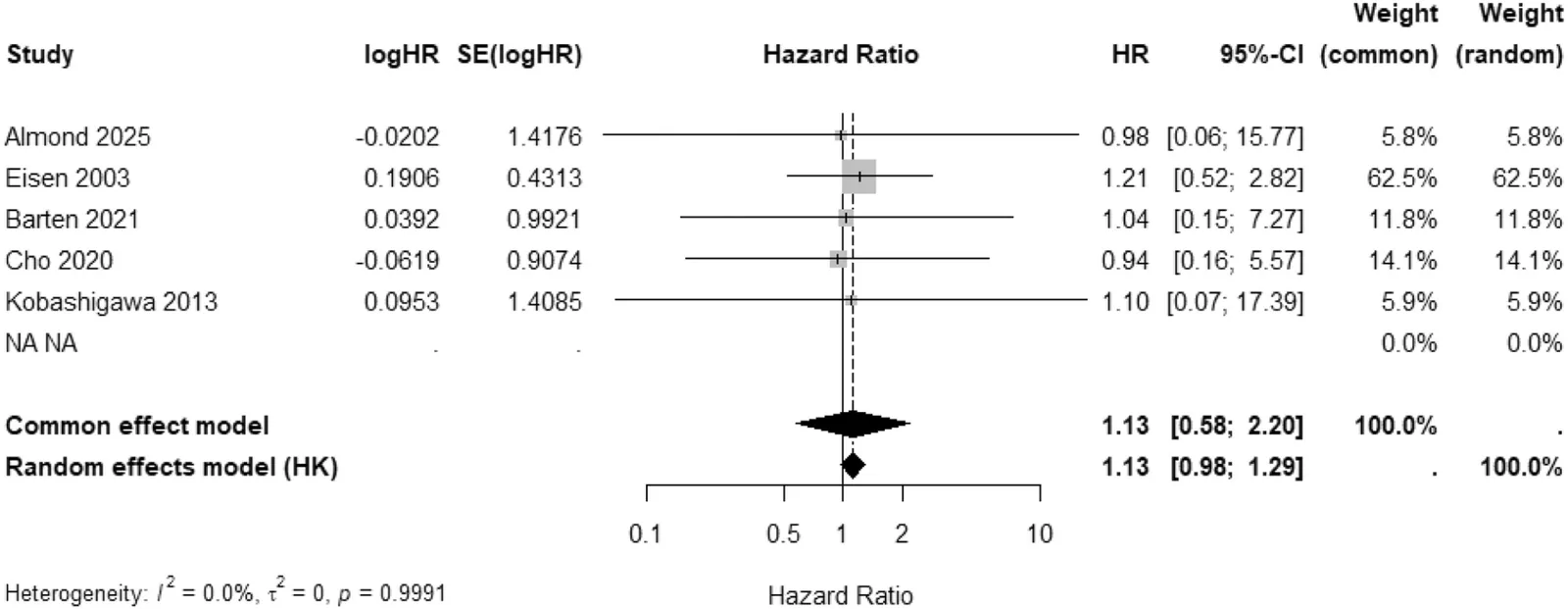
Forest plot – All cause mortality.

**Figure 3:**
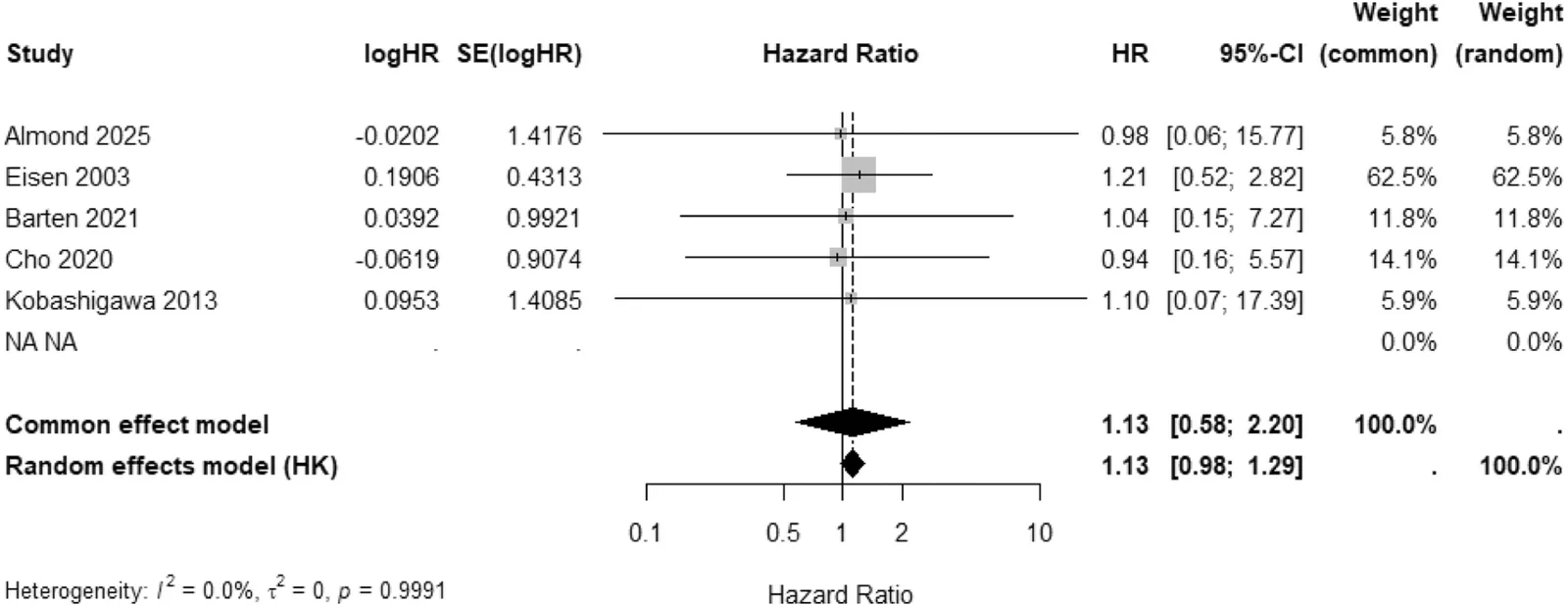
Forest plot – Acute rejection.

**Figure 4:**
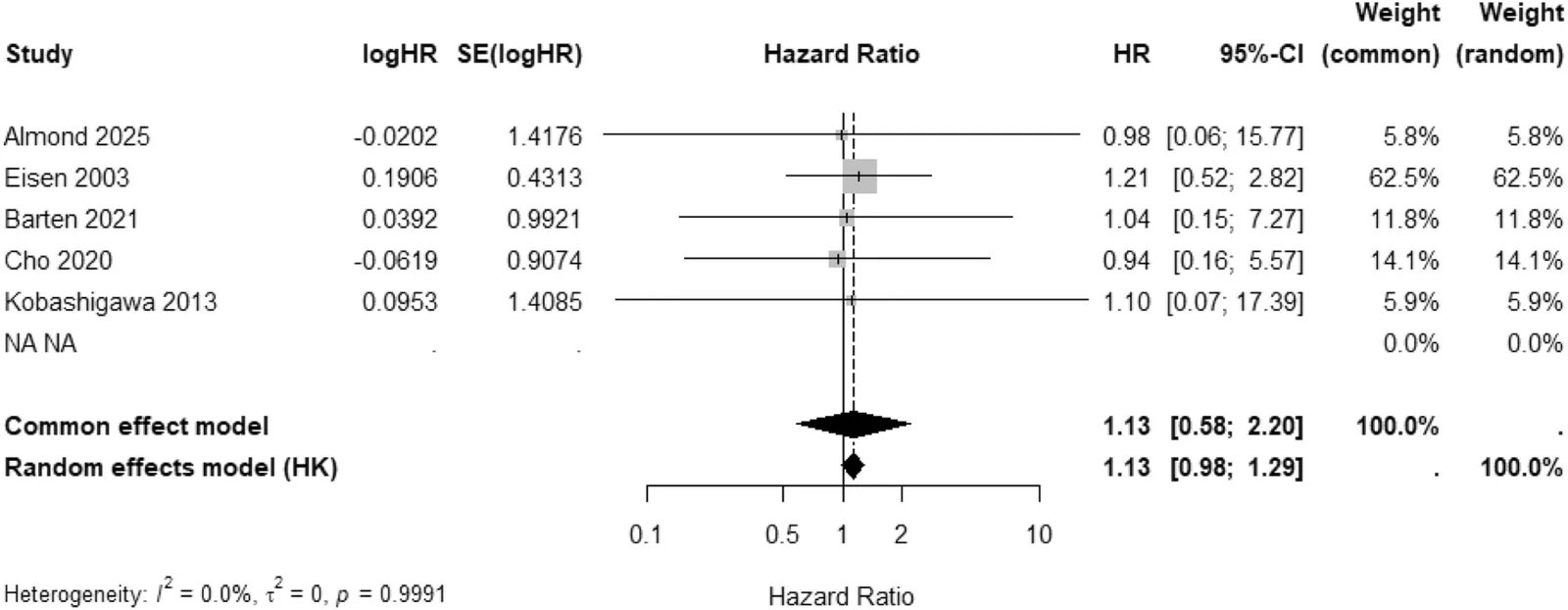
MATE-6.

**Figure 5:**
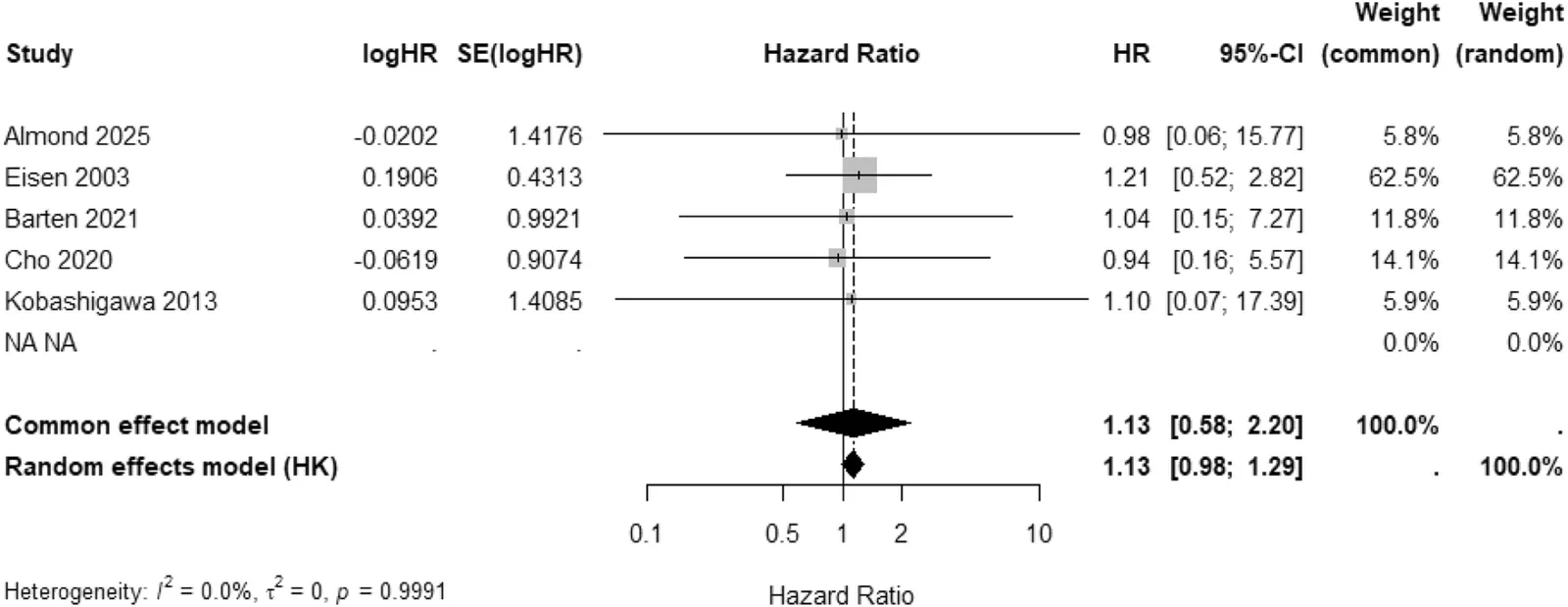
MATE-3.

**Figure 6:**
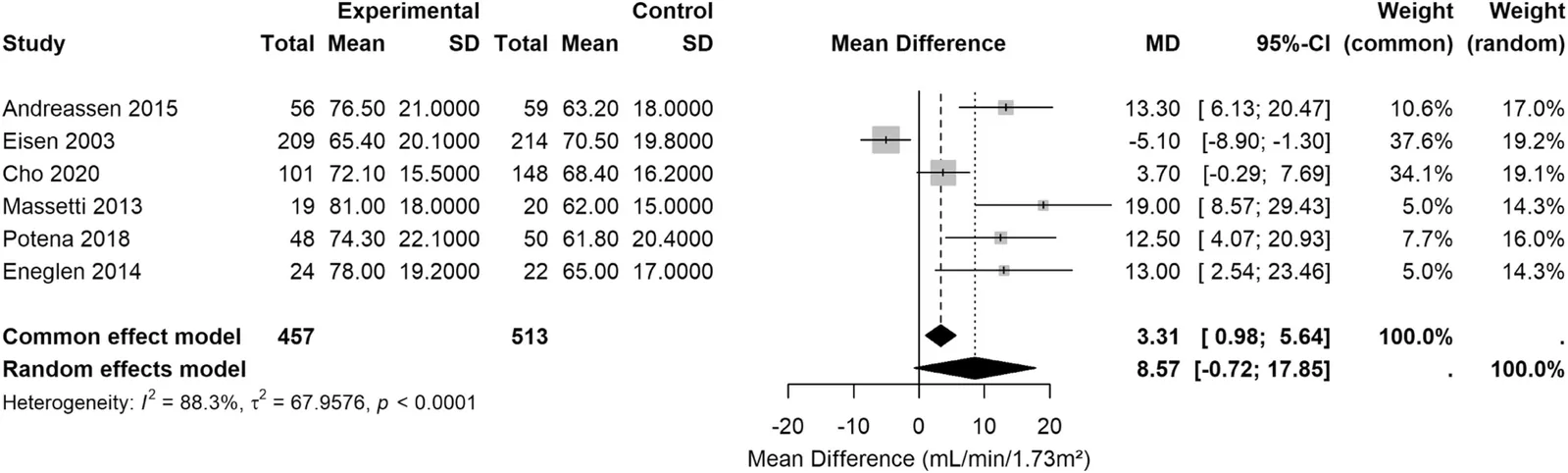
Renal function.

**Figure 7:**
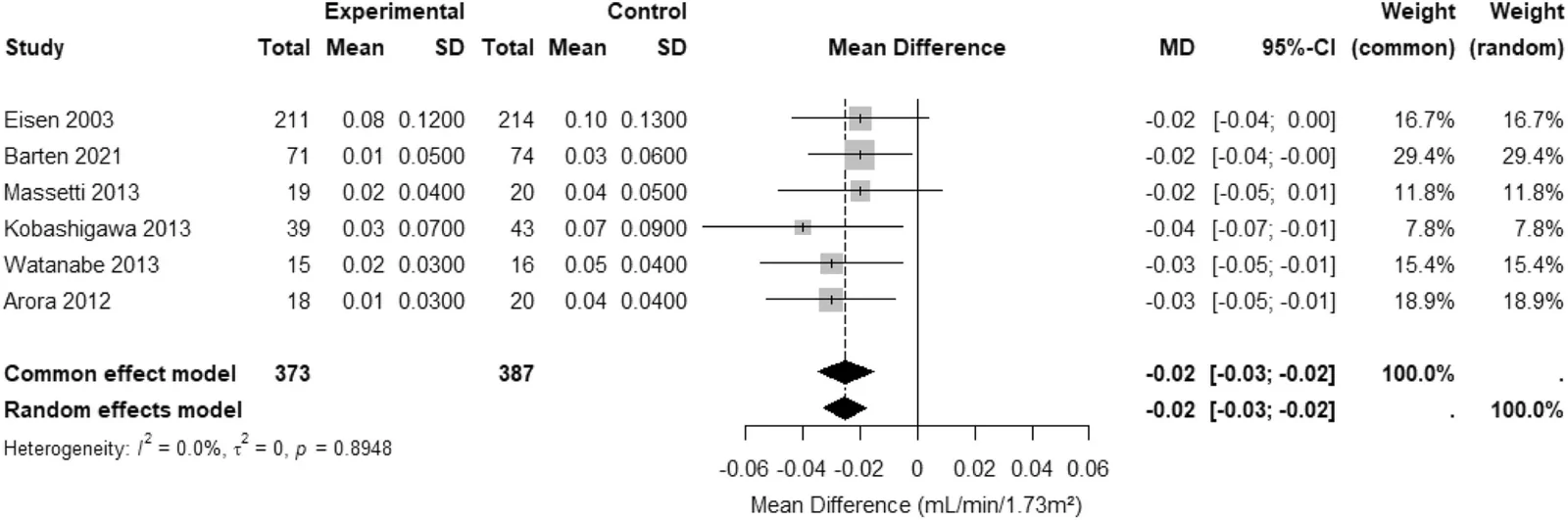
IVUS.

**Figure 8:**
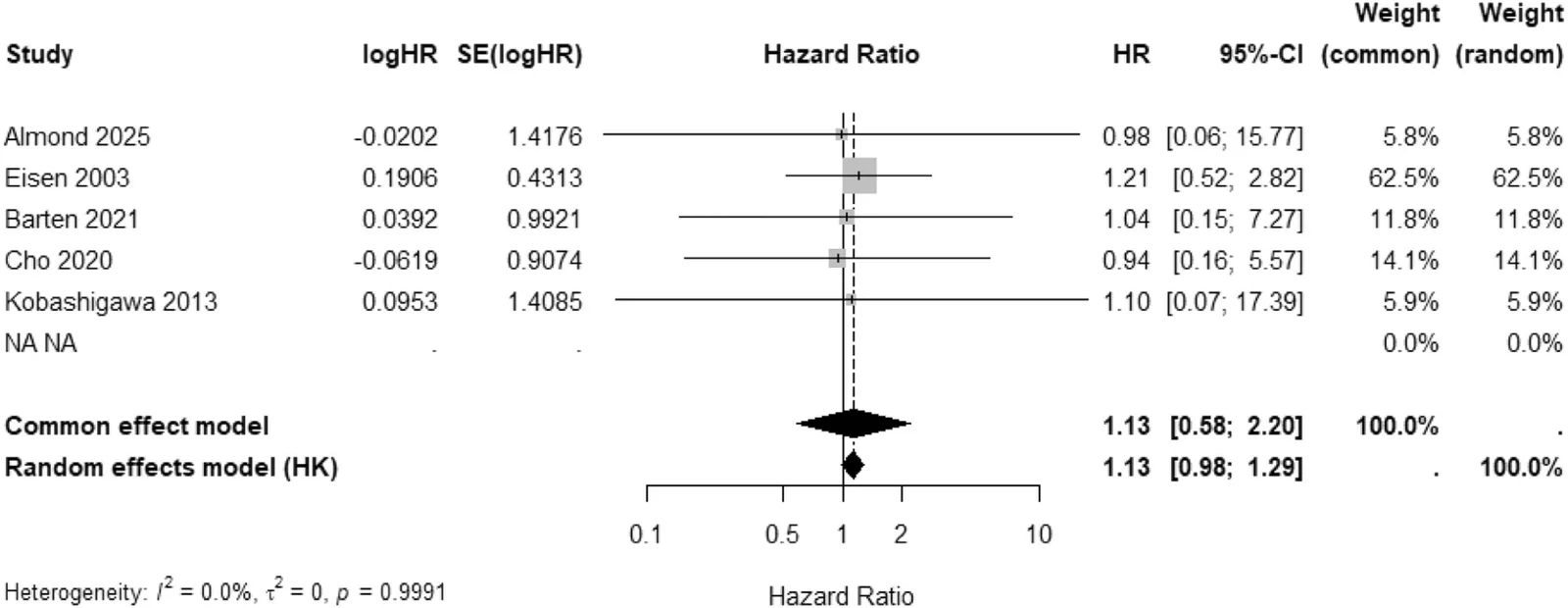
Survival.

**Figure 9:**
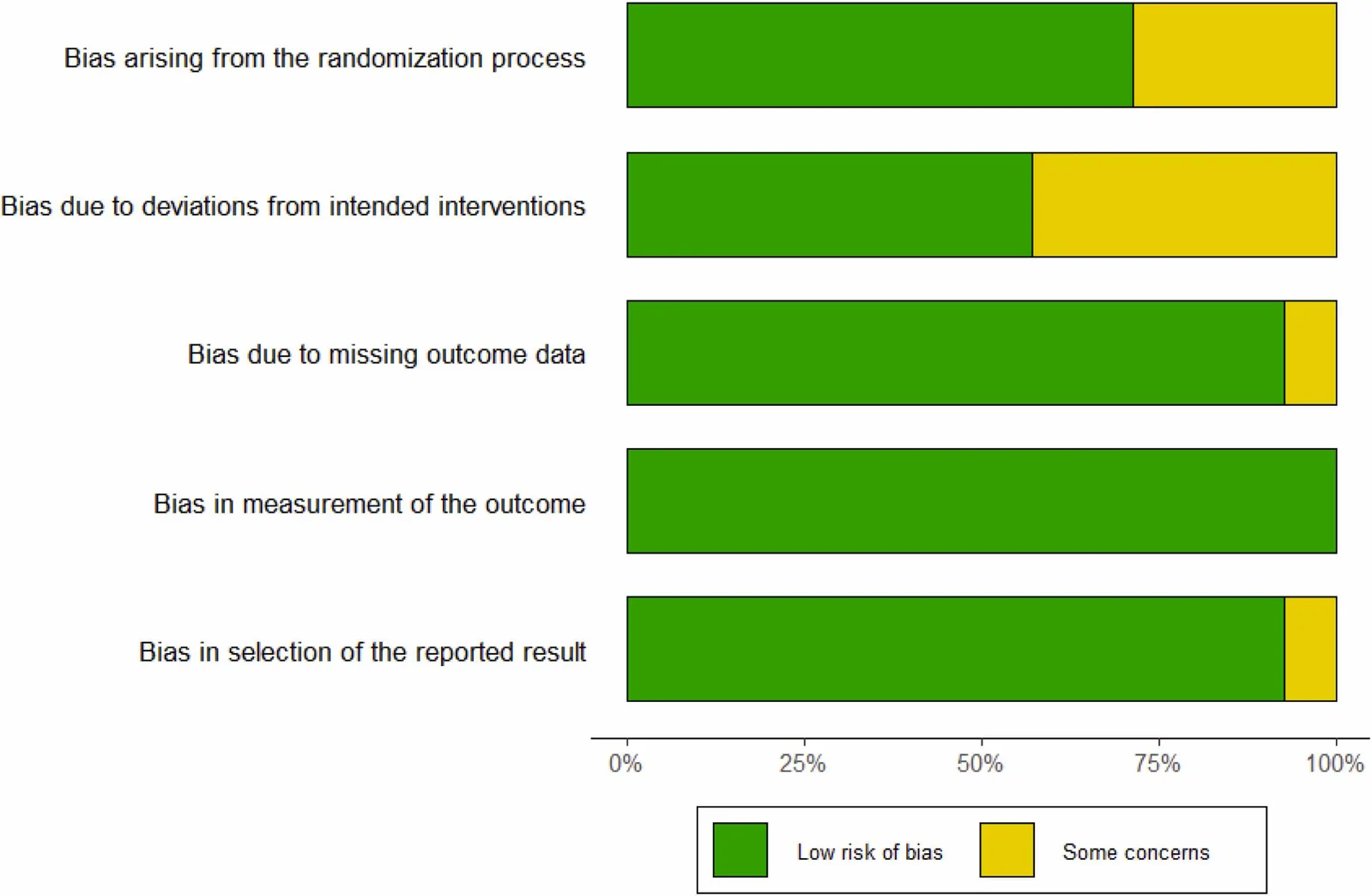
Risk of Bias.

**Figure 10:**
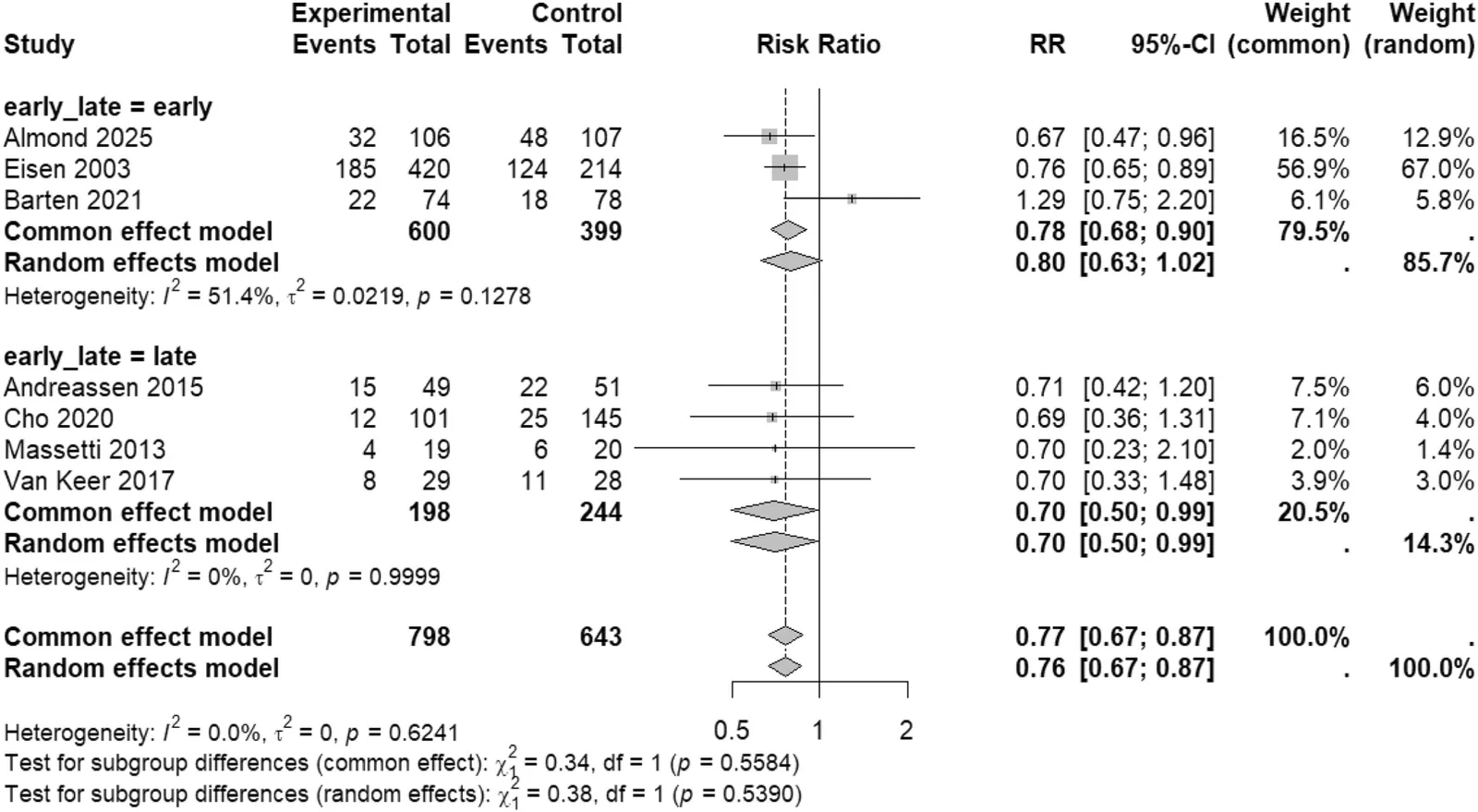
Early vs Late introduction of EVR – MATE-3.

**Figure 11:**
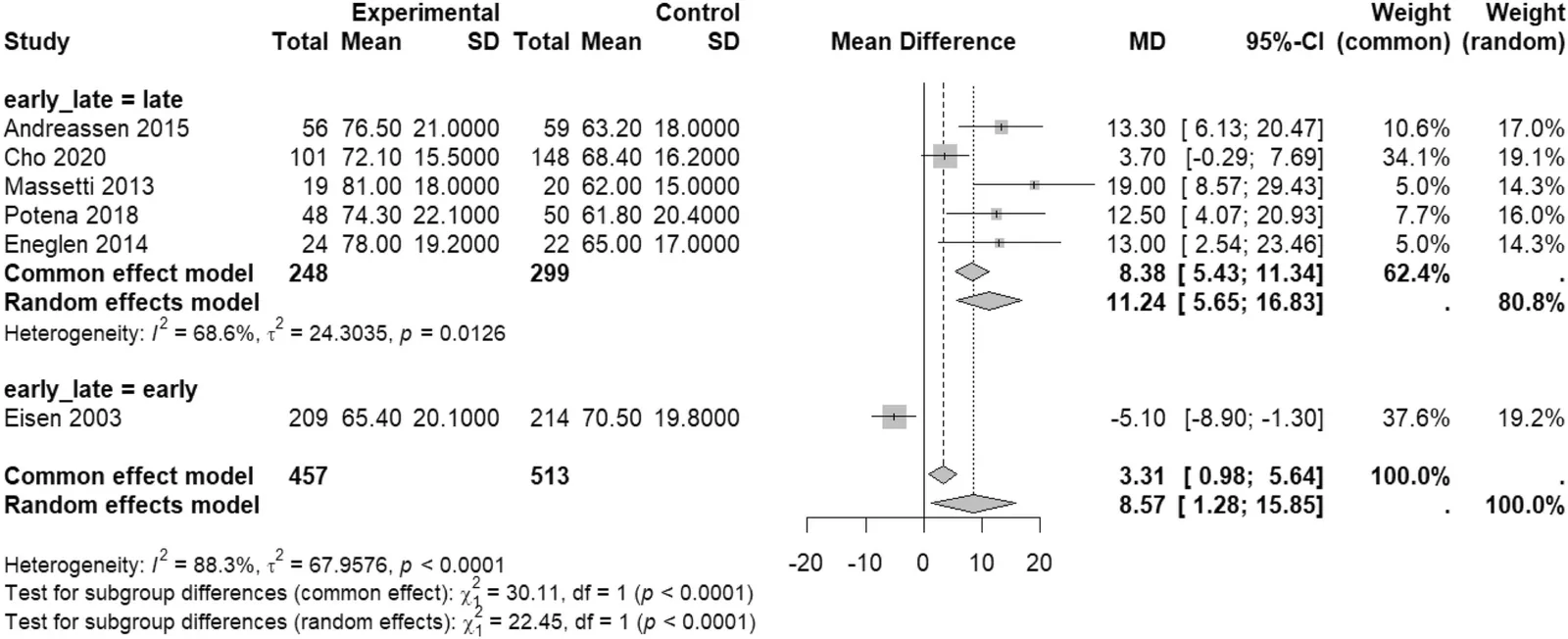
Early vs Late introduction of EVR – Renal function.

**Figure 12:**
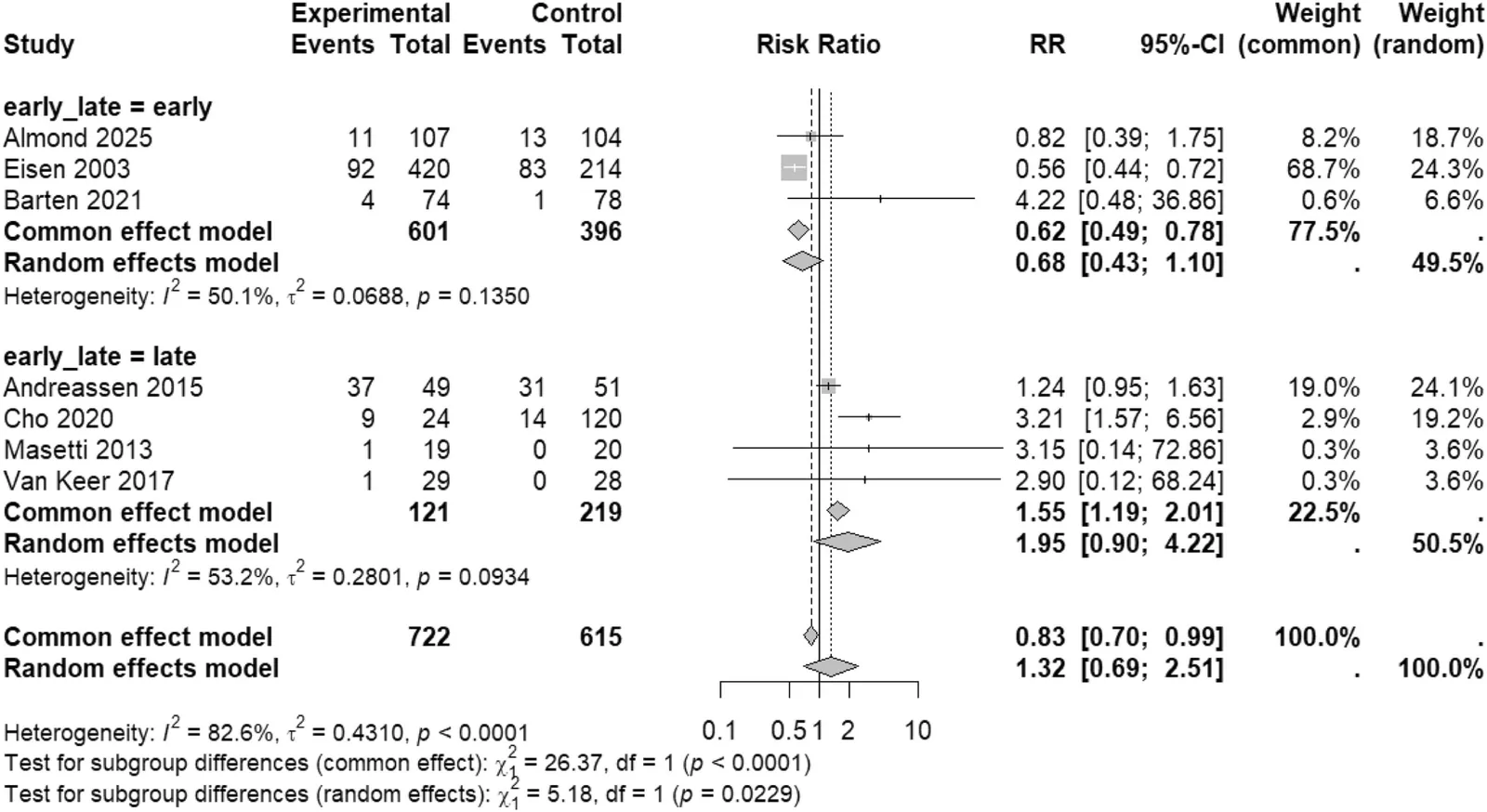
Early vs Late introduction of EVR – Acute rejection.

**Figure 13:**
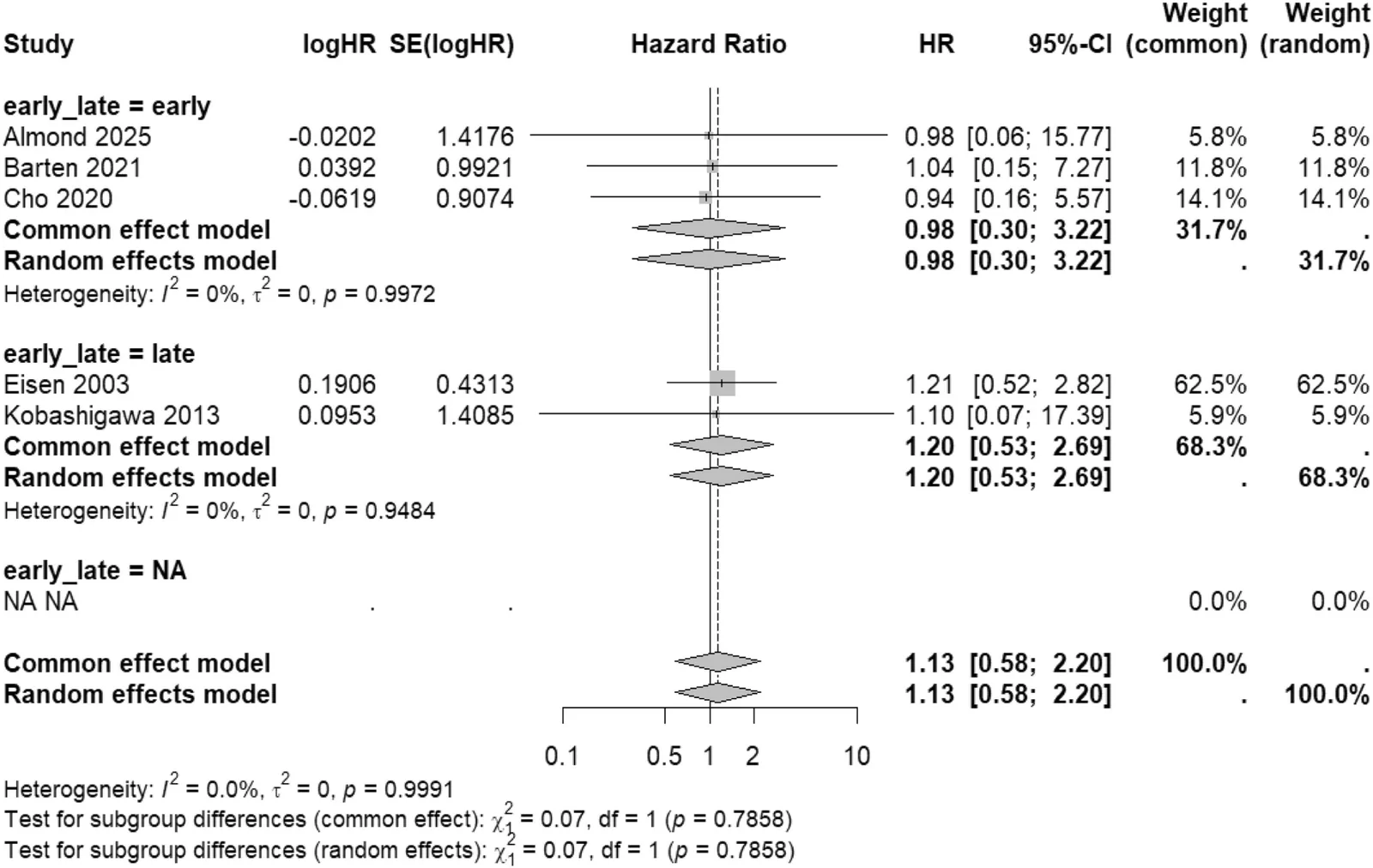
Early vs Late introduction of EVR – Survival.

### Eligibility criteria

Studies were included if they met all of the following criteria^1^: enrolled heart transplant recipients (adult and/or pediatric populations, with extractable pediatric data when applicable)^2^; evaluated mTOR inhibitor–based regimens (everolimus only, alone or combined with reduced-dose tacrolimus)^3^; included a comparator group receiving standard immunosuppressive therapy (tacrolimus with or without mycophenolate mofetil or equivalent non-mTOR regimens)^4^; reported at least one outcome of interest^10^; were randomized controlled trials or comparative observational cohort/registry studies. Studies were excluded if they included studies without a comparator group; mixed-organ transplant studies without separable heart transplant data; conference abstracts with insufficient data. No restrictions on publication date, language, or follow-up duration were applied.

### Search strategy and screening process

A comprehensive literature search was performed in MEDLINE/PubMed, EMBASE, and the Cochrane Central Register of Controlled Trials (CENTRAL) from inception to the final search date. The search strategy combined controlled vocabulary and free-text terms related to heart transplantation, mTOR inhibitors (everolimus or sirolimus), calcineurin inhibitors, and immunosuppressive therapy. However, even though search terms included both everolimus and sirolimus to maximize sensitivity, no eligible sirolimus studies met criteria for quantitative synthesis and all analyses were restricted to everolimus-based regimens.The full search strategies for each database are provided in Supplementary Methods.

After removal of duplicates, titles and abstracts were independently screened by two reviewers (B. and S.), followed by full-text assessment of potentially eligible studies. Disagreements were resolved by consensus or consultation with a third reviewer.

### Endpoints and sub analysis

The primary outcome was the MATE-3 composite score, a validated ordinal endpoint reflecting the cumulative burden and severity of three major adverse transplant events: acute cellular rejection (ACR), cardiac allograft vasculopathy (CAV), and chronic kidney disease (CKD), using definitions adapted from international consensus criteria.

Secondary outcomes included individual components of MATE-3, renal function parameters, mortality or graft loss, adverse events, and drug pharmacokinetics. Safety outcomes were additionally summarized using broader adverse event composites (MATE-6, when available).

Because concomitant calcineurin inhibitor exposure varied substantially across studies, immunosuppressive strategy (full-dose CNI, reduced-dose CNI, or CNI withdrawal) was recorded and considered during qualitative interpretation of pooled results.

### Risk of bias assessment

Risk of bias was assessed using the Cochrane Risk of Bias 2.0 tool for randomized trials and ROBINS-I for observational studies. Two reviewers conducted assessments independently by BN and SHV, with disagreements resolved through discussion.

Small-study effects and publication bias were evaluated using funnel plots and Egger’s test when at least ten studies were available for a given outcome.

A leave-one-out sensitivity analysis was conducted sequentially excluding each study to evaluate the robustness of pooled estimates and to identify influential studies.

### Statistical analysis

Meta-analyses were performed using random-effects models (restricted maximum likelihood estimation) to account for anticipated clinical and methodological heterogeneity. Effect estimates were expressed as risk ratios (RR) or odds ratios (OR) for dichotomous outcomes and mean difference (MD) or standardized mean difference (SMD) for continuous outcomes, each with corresponding 95% confidence intervals. Heterogeneity was assessed using Cochran’s Q test and quantified with the I² statistic.

Prespecified subgroup analyses were conducted to explore sources of clinical heterogeneity and effect modification according to baseline and treatment-related characteristics. Subgroups included age at baseline (<85 vs ≥85 years), sex, race, pediatric versus adult recipients (≥18 years), body mass index or weight, baseline creatinine clearance, time since transplantation (early ≤12 months vs late >12 months), history of prior rejection (yes vs no), indication for transplantation (including congenital heart disease subtypes), graft type (primary transplant vs retransplantation), immunosuppressive regimen (mTOR-based intervention vs standard control), everolimus exposure (high vs low dose), use of induction therapy (yes vs no), and immunosuppressive strategy (de novo initiation vs conversion therapy). Interaction tests were performed when applicable to assess statistical differences between subgroups. Subgroup findings were considered exploratory and interpreted cautiously.

### Survival analysis

For time-to-event outcomes, individual patient data (IPD) were reconstructed from published Kaplan–Meier curves using established digitalization and reconstruction methods. Survival probabilities were extracted at predefined time points, and numbers at risk and total events were incorporated when available to improve accuracy of the reconstructed datasets. The reconstructed survival data were internally validated by visually comparing estimated curves with the original published Kaplan–Meier plots.

A one-stage meta-analytic approach was applied using Cox proportional hazards regression to estimate pooled hazard ratios (HRs) and corresponding 95% confidence intervals. Study-level clustering was accounted for by including study as a random effect. The proportional hazards assumption was evaluated using Schoenfeld residuals and graphical inspection. Sensitivity analyses included leave-one-out procedures and exclusion of studies at high risk of bias.

All statistical analyses were made using R Studio 4.4.3 (R Foundation for Statistical Computing, Vienna, Austria) using packages “meta”.^4^

### MATE-3 and MATE-6^10^

Clinical efficacy and safety were assessed using validated composite endpoints known as Major Adverse Transplant Events (MATE), as defined by the TEAMMATE trial protocol.^10^ The primary efficacy endpoint, MATE-3, was defined as the time to the first occurrence of any of the following events: biopsy-proven acute rejection (cellular rejection grade ≥2R or antibody-mediated rejection grade ≥1), evidence of cardiac allograft vasculopathy (CAV) detected via angiography or intravascular ultrasound (IVUS), or the development of chronic kidney disease (defined as a sustained reduction in estimated glomerular filtration rate [eGFR] or the need for renal replacement therapy). To provide a broader assessment of clinical stability and drug tolerability, we also analyzed the MATE-6 endpoint. This composite outcome includes all elements of MATE-3 plus three additional safety-related criteria: the occurrence of serious drug-related adverse events (such as severe non-viral infections, cytopenias, or pneumonitis), definitive study drug discontinuation due to intolerance or toxicity, and all-cause mortality.

## Results

### Study selection and eligibility criteria

A comprehensive systematic search of MEDLINE/PubMed, EMBASE, and the Cochrane Library identified 2411 records. After removal of 501 duplicates, 1910 unique citations were screened by title and abstract. Of these, 1901 records underwent initial eligibility assessment. Following independent review and resolution of discrepancies between 2 investigators, 125 studies were retained for detailed evaluation. Ultimately, 60 studies were included in the final qualitative and quantitative synthesis, comprising 12 full-text publications and 48 studies with extractable data from published abstracts. Because outcome availability differed substantially across studies, each pooled analysis included only the subset of studies reporting extractable data for the specific endpoint of interest. Consequently, the number of studies contributing to individual meta-analyses varied across outcomes, ranging from five to six studies for some endpoints and larger numbers for others.

### All cause mortality

The pooled analysis showed no statistically significant difference in all-cause mortality between everolimus-based regimens and standard immunosuppression (RR 0.98–1.00), with minimal between-study heterogeneity. The Eisen et al. (2003) trial contributed most of the statistical weight and was the only study suggesting a numerical increase in mortality with everolimus.^11^ More recent studies did not demonstrate this pattern. Subgroup analyses suggested a more favorable survival pattern with conversion strategies compared with de novo initiation, and with later rather than early introduction, although these differences were not statistically significant.

### Acute rejection

Acute rejection outcomes were inconsistent across studies. The fixed-effect model suggested a modest reduction in rejection risk with everolimus (RR 0.83, 95% CI 0.70–0.99), but this effect was not maintained under the random-effects model (RR 1.32, 95% CI 0.69–2.51), reflecting substantial heterogeneity between trials. Subgroup analyses provided further insight: de novo initiation of everolimus was associated with a significant reduction in acute rejection (RR 0.67, 95% CI 0.54–0.84), whereas conversion strategies showed a non-significant increase in risk (RR 1.22, 95% CI 0.95–1.57). Timing appeared particularly important, with early introduction associated with lower rejection rates (RR 0.62, 95% CI 0.49–0.78), while late conversion was linked to increased risk (RR 1.55, 95% CI 1.19–2.01). The largest trial, Eisen et al. (2003), which contributed most of the weight, demonstrated a clear reduction in acute rejection (RR 0.56, 95% CI 0.44–0.72) when everolimus was combined with full-dose calcineurin inhibition, whereas smaller studies with aggressive CNI withdrawal reported higher rejection rates.^11^

### MATE-6

The pooled MATE-6 analysis favored the control arm, indicating a higher burden of safety-related events in recipients receiving everolimus-based regimens. Because individual components of MATE-6 were not consistently reported across studies, the specific adverse events driving this result could not be formally quantified.

### MATE-3

The composite endpoint favored everolimus (RR 0.76, 95% CI 0.67–0.87), with no significant heterogeneity (I²=0%)., driven primarily by reductions in cardiac allograft vasculopathy and preservation of renal function. Although acute rejection rates varied across strategies, the benefits in vascular and renal outcomes consistently shifted the overall composite toward everolimus. Subgroup analyses showed the greatest benefit with de novo initiation, likely reflecting early suppression of vasculopathic processes, while conversion strategies demonstrated benefit mainly through renal preservation. Pediatric recipients also showed favorable outcomes, largely due to reduced CAV progression and sustained renal function. Moderate heterogeneity across studies likely reflects differences in definitions of renal dysfunction and methods used to assess CAV.

### Cardiac allograft vasculopathy

Six studies comprising 760 heart transplant recipients (373 receiving everolimus and 387 receiving standard immunosuppression) evaluated CAV progression using intravascular ultrasound (IVUS). Across studies, everolimus was associated with significantly lower progression of intimal thickening compared with control therapy (MD −0.02, 95% CI −0.03 to −0.02). No statistical heterogeneity was observed (I² = 0%, p = 0.89), and the pooled estimate was identical in both common-effect and random-effects models. Individual study estimates consistently favored everolimus, with statistically significant reductions observed in Kobashigawa et al., Watanabe et al., and Arora et al., while Eisen et al., Barten et al., and Massetti et al. demonstrated a similar direction of effect with smaller magnitudes. Barten et al. contributed the largest statistical weight (29.4%), followed by Arora et al. (18.9%), Eisen et al. (16.7%), and Watanabe et al. (15.4%).

### Renal function

Renal outcomes demonstrated marked heterogeneity across studies (I² >70%). In random-effects models, everolimus was associated with improved eGFR compared with standard therapy, with pooled mean differences ranging from +10 to +15 mL/min in conversion and CNI-minimization protocols. Registry data (Cho 2020) showed more modest but directionally consistent improvements. Results varied substantially by CNI exposure strategy. Trials combining everolimus with full-dose cyclosporine (Eisen 2003) favored the control arm, whereas studies employing CNI minimization or withdrawal (SCHEDULE, Potena, and Engelen) consistently favored everolimus.

### Early versus late everolimus introduction

Across included studies, early initiation generally referred to introduction within the first year after transplantation, with several trials initiating everolimus during the first weeks or months after surgery. Late-conversion studies typically enrolled stable recipients more than one year after transplantation and introduced everolimus primarily to facilitate CNI minimization or address renal dysfunction.

Subgroup analyses according to timing of everolimus initiation demonstrated significant effect modification for acute rejection and renal function. For acute cellular rejection, early initiation (<12 months) was associated with a lower risk of rejection (RR 0.68, 95% CI 0.43–1.10), whereas late-conversion (>12 months) strategies showed a trend toward increased rejection (RR 1.95, 95% CI 0.90–4.22), with a significant subgroup interaction (p = 0.023).

Renal outcomes demonstrated the opposite pattern. Late-conversion protocols were associated with significantly greater preservation of renal function (MD 11.24 mL/min, 95% CI 5.65–16.83), whereas studies initiating everolimus early showed no consistent renal benefit. The difference between subgroups was highly significant (p < 0.0001).

In contrast, no significant subgroup differences were observed for all-cause mortality (p = 0.19) or the composite safety endpoint MATE-6 (p = 0.30), suggesting that the impact of timing is primarily related to rejection risk and renal preservation rather than overall survival or treatment-related adverse events.

### Risk of bias

Eleven RCTs were assessed using the Cochrane Risk of Bias 2.0 (RoB 2) tool, one cohort study was evaluated with ROBINS-I. Overall methodological quality among the RCTs was high. Eight trials were rated at low risk of bias (SCHEDULE, TEAMMATE, MANDELA, CERTICAN, TRANSFORM, Stypmann 2015^8^, NOCTET, and Massetti 2013^5^); six were rated as having some concerns (SHIRAKISHI, RAD-TAC, CECERE/Cercari, Chou 2007^6^, Watanabe 2013^7^, and Schweiger 2006^9^). Concerns were driven primarily by deviations from intended interventions, largely attributable to open-label designs, and incomplete reporting of allocation concealment. Randomization, missing outcome data, outcome measurement, and selective reporting were satisfactorily addressed in most trials. The cohort study was rated at moderate-to-high risk of bias due to potential confounding and selection bias, consistent with its non-randomized design. Overall, the body of evidence was predominantly at low risk of bias, with most limitations reflecting transparency rather than fundamental methodological flaws.

### Sensitivity analysis

Leave-one-out sensitivity analyses demonstrated that pooled estimates were generally robust. For IVUS-defined cardiac allograft vasculopathy, exclusion of a single study attenuated the pooled effect and resulted in loss of statistical significance, indicating some sensitivity to individual-study influence. For all remaining outcomes, sequential exclusion of individual studies did not materially alter pooled estimates or statistical conclusions.

## Discussion

In this updated systematic review and meta-analysis, 60 studies met eligibility criteria; however, the primary quantitative analyses were derived from 12 full-text comparative studies comprising 1786 heart transplant recipients with sufficient data for pooled outcome assessment. Five principal findings emerged. First, everolimus provides significant vasculoprotection, reducing the progression of cardiac allograft vasculopathy (CAV). Second, when combined with calcineurin inhibitor (CNI) minimization, it functions as an effective strategy for renal preservation. Third, the risk of acute rejection appears highly dependent on the timing and intensity of CNI withdrawal. Fourth, the composite clinical endpoint MATE-3 favors everolimus, reflecting the combined benefits on vascular and renal outcomes. Finally, these advantages are counterbalanced by higher rates of drug-related toxicity and treatment discontinuation captured in the MATE-6 safety composite.

The vascular protective effect observed in this analysis represents one of the most clinically relevant findings. Beyond its immunosuppressive activity, everolimus exerts antiproliferative effects on endothelial cells and vascular smooth muscle cells, limiting the intimal hyperplasia that underlies CAV.^12^ Our pooled IVUS analysis demonstrated a significant attenuation of intimal thickening, reinforcing the biological rationale for mTOR inhibition as a strategy to modify the natural history of transplant vasculopathy. Given that CAV remains one of the leading causes of late graft failure and mortality after heart transplantation,^2^ early pharmacologic strategies targeting vascular remodeling may have important long-term implications for graft longevity.

Renal preservation represents another central advantage of everolimus-based regimens. Long-term exposure to CNIs is intrinsically associated with progressive nephrotoxicity characterized by arteriolar hyalinosis, interstitial fibrosis, and glomerulosclerosis.^13^, ^14^ In our analysis, strategies incorporating everolimus with reduced CNI exposure were consistently associated with improved renal function compared with conventional regimens. These findings align with previous trials by Wang et al. and Gullestad et al.,^15^ and suggest that everolimus may play a key role in mitigating chronic CNI-related kidney injury. Importantly, the magnitude of renal benefit appears to depend on the timing of intervention.^14^ Early introduction as part of a structured CNI minimization strategy was associated with the greatest preservation of eGFR, whereas conversion in patients with advanced renal dysfunction resulted in more modest improvement. This observation supports the concept that prevention of CNI-induced nephrotoxicity is likely more achievable than reversal once structural damage has occurred.

Acute rejection remains a major concern when modifying standard immunosuppressive regimens.^1^, ^4^

An additional explanation for this heterogeneity may be an era effect. The included studies span more than two decades of heart transplantation practice, during which substantial changes occurred in donor selection, perioperative management, rejection surveillance, and maintenance immunosuppressive protocols. Earlier trials frequently evaluated everolimus in combination with full-dose cyclosporine, whereas more contemporary studies incorporated structured CNI minimization strategies. Therefore, part of the observed variability may reflect changes in transplant practice over time rather than the isolated effect of everolimus itself.

Our findings indicate that the risk associated with everolimus is strongly protocol dependent. De novo strategies combining everolimus with controlled CNI exposure generally maintained adequate immunologic protection, whereas late conversion protocols involving abrupt or aggressive CNI withdrawal were associated with higher rejection rates. This pattern suggests that everolimus should not be interpreted as a direct substitute for calcineurin inhibition, but rather as a complementary agent within carefully balanced immunosuppressive strategies. These findings emphasize the importance of maintaining sufficient baseline immunosuppression when transitioning to mTOR-based regimens.

When evaluated through the composite MATE-3 endpoint,^10^ which integrates rejection, CAV, and renal dysfunction, everolimus demonstrated an overall favorable clinical profile. This composite outcome captures the cumulative burden of major adverse transplant events and provides a more integrated assessment of graft health than isolated endpoints.^10^ In our analysis, the reduction in CAV progression and preservation of renal function outweighed the variability observed in rejection rates, resulting in a net composite benefit favoring everolimus. These findings support the concept that long-term graft outcomes depend on balancing immunologic control with protection from chronic organ toxicity.

The less favorable MATE-6 profile observed with everolimus likely reflects the well-recognized adverse-effect spectrum of mTOR inhibitors, including impaired wound healing, edema, oral ulcers, proteinuria, cytopenias, and noninfectious pneumonitis, which have been consistently reported in previous heart-transplant studies.^16^, ^17^ However, because individual components of MATE-6^10^ were not uniformly reported across studies, the relative contribution of specific adverse events could not be formally quantified in the present meta-analysis.

The absence of a mortality difference between treatment strategies deserves careful interpretation. While our meta-analysis did not detect a survival advantage for everolimus, most included studies had relatively short follow-up durations (typically 12–24 months), a timeframe that may be insufficient to capture the long-term consequences of CAV progression. Because transplant vasculopathy evolves gradually over several years,^1^ the vascular protection observed in IVUS studies may translate into survival benefits only with longer follow-up. At the same time, increased drug-related toxicity and discontinuation may partially offset potential long-term advantages, contributing to the neutral mortality signal observed across randomized trials.

Subgroup analyses suggest that the apparent effect of early versus late everolimus initiation is driven less by timing itself and more by the accompanying immunosuppressive strategy. For acute rejection, early-initiation protocols generally maintained full or near-full calcineurin inhibitor (CNI) exposure and were associated with lower rejection rates, whereas late-conversion studies frequently incorporated substantial CNI minimization or withdrawal and demonstrated less favorable rejection outcomes.^18^, ^11^, ^19^ These findings indicate that the observed differences are likely attributable to variations in baseline immunosuppressive intensity rather than to the chronological timing of everolimus introduction alone. Accordingly, everolimus appears to be most effective when integrated into balanced immunosuppressive regimens that preserve adequate CNI coverage while avoiding excessive nephrotoxic exposure.

In contrast, the beneficial effects of everolimus on cardiac allograft vasculopathy and renal function were observed across a broader range of initiation strategies. Studies introducing everolimus early after transplantation consistently demonstrated attenuation of intimal thickening, supporting a preventive role in vascular remodeling, whereas late-conversion protocols primarily derived benefit through CNI minimization and renal preservation.^11^, ^20^ Collectively, these findings suggest that everolimus provides its greatest clinical value when used as part of a CNI-sparing strategy, allowing preservation of graft vasculature and kidney function without compromising overall survival. The favorable MATE-3 profile observed across studies further supports this integrated benefit despite variability in individual outcome components.^3^, ^18^

Wound-healing complications remain an important consideration when initiating everolimus early after transplantation. Although reporting was inconsistent across studies and precluded formal quantitative synthesis, available evidence suggests that major wound complications were relatively uncommon and largely confined to the early postoperative period.^18^, ^19^ In the dedicated analysis by Barten et al.^20^, overall wound-complication rates were 14.8% with everolimus, 13.1% with azathioprine, and 8.4% with mycophenolate mofetil, while wound and sternal dehiscence occurred in fewer than 2% of patients across treatment groups. Notably, complication rates were higher when everolimus was initiated within the first 24 h after transplantation compared with initiation between 48 and 72 h postoperatively, suggesting that very early exposure may interfere with surgical healing. In contrast, studies evaluating late-conversion strategies reported few transplant-related wound-healing events, indicating that this risk is substantially attenuated once surgical healing has been completed. Therefore, concerns regarding wound healing should primarily influence the timing of early postoperative initiation rather than preclude the use of everolimus altogether.

## Study limitations

This meta-analysis has several limitations. Substantial clinical and methodological heterogeneity was observed across trials, driven by differences in everolimus initiation timing, calcineurin inhibitor (CNI) minimization strategies, and patient risk profiles. Random-effects models and prespecified subgroup analyses were employed to partially account for this variability. Several studies were open-label and carried some risk of bias, particularly related to deviations from intended interventions, and risk of bias was formally assessed using RoB 2 and ROBINS-I. Safety estimates were influenced by older protocols combining everolimus with full-dose CNI exposure, which are not representative of contemporary clinical practice. Follow-up duration was relatively short in most studies (typically 12–24 months), limiting conclusions regarding long-term survival and chronic graft outcomes. Some subgroup analyses, including pediatric cohorts and late-conversion protocols, were based on small sample sizes or single studies and should therefore be considered hypothesis-generating. In addition, subgroup analyses evaluating early versus late everolimus initiation should be interpreted cautiously, as differences in concomitant immunosuppressive strategies, particularly CNI exposure, may confound the isolated effect of treatment timing. Observational studies contributed to renal and survival analyses and remained susceptible to residual confounding despite propensity adjustment. Finally, composite endpoints such as MATE-3 combine heterogeneous clinical domains, which may obscure differential effects on individual components. These limitations were addressed where possible through sensitivity analyses, subgroup stratification, and leave-one-out analyses, the full results of which are available in the Supplemental Material.

## Conclusion

Taken together, our findings suggest that everolimus should be viewed not simply as an alternative immunosuppressive agent, but as a strategic tool for long-term graft preservation. Everolimus-based regimens were associated with reduced progression of cardiac allograft vasculopathy, improved renal function when coupled with CNI minimization, and a favorable MATE-3 profile without compromising overall survival. Importantly, subgroup analyses indicate that clinical outcomes are driven less by the timing of everolimus initiation itself and more by how the drug is integrated into the overall immunosuppressive regimen. The greatest benefit appears to be achieved when everolimus is used to facilitate CNI minimization while maintaining adequate immunologic protection. Accordingly, recipients at increased risk for CAV, CNI-related nephrotoxicity, or prolonged CNI exposure may derive the greatest benefit from this strategy. Future studies with longer follow-up are needed to determine whether the observed vascular and renal advantages ultimately translate into improved long-term graft and patient survival.

## Financial Disclosure Statement

The authors declare that no specific funding was received for this study. This research did not receive any grant from funding agencies in the public, commercial, or not-for-profit sectors.

## Search strategies of each database

PUBMED: (“Heart transplantation” OR “Grafting, Heart” OR “Graftings, Heart” OR “Heart graftings” OR “Transplantation, Cardiac” OR “Transplantation, Heart” OR “Heart Transplantations” OR “Transplantations, Heart” OR “Cardiac Transplantation” OR “Cardiac Transplantations” OR “Transplantations, Cardiac”) AND (Everolimus OR "Mammalian target of rapamycin inhibitors" OR "mTOR inhibitors") AND (“Immunosuppressive Agents” OR “Agents, Immunosuppressive” OR “Immunosuppressive Agent” OR “Agent, Immunosuppressive” OR Immunosuppressant OR Immunosuppressants OR ”Mycophenolate Mofetil” OR “Calcineurin Inhibitor”).

Embase: Heart transplantation AND everolimus AND calcineurin inhibitor.

Cochrane: Heart transplantation AND everolimus AND calcineurin inhibitor.

## Declaration of Competing Interest

The authors declare that they have no known competing financial interests or personal relationships that could have appeared to influence the work reported in this paper.
